# Evaluation of Micronuclei and Cytomorphometric Changes in Patients with Different Tobacco Related Habits Using Exfoliated Buccal Cells

**DOI:** 10.31557/APJCP.2021.22.6.1851

**Published:** 2021-06

**Authors:** Sivakumar Kokila, Harikrishnan Prasad, Muthusamy Rajmohan, Kenniyan Kumar Srichinthu, Loganathan Mahalakshmi, Sivanandham Shanmuganathan, Perumal Prema

**Affiliations:** *Department of Oral and Maxillofacial Pathology, KSR Institute of Dental Science and Research, Tiruchengode, Namakkal, India. *

**Keywords:** Micronuclei, cytomorphometry, tobacco, cytoplasmic area, Feulgen, exfoliated buccal cells

## Abstract

**Background:**

Tobacco is one of the main reasons behind the occurrence of oral cancer. Oral cancer, even though being the tenth most common cancer in the world, gets diagnosed at an advanced stage and ends up with poor prognosis. So early diagnosis is the need of the hour. Our study aimed to evaluate the genotoxic changes in patients with different tobacco habits using buccal exfoliated cells.

**Methods:**

Buccal smears were taken from smokers (30), smokeless tobacco users (30), combined tobacco users (30) and controls (30) with clinically normal oral mucosa. All the smears were stained with Papanicolaou stain and Feulgen stain and viewed under light microscope for the evaluation of mean number of micronuclei, mean micronuclei per cell, frequency of cells showing micronuclei, nuclear area, cytoplasmic area, nuclear-cytoplasmic ratio.

**Results:**

Mean number of micronuclei, mean micronuclei per cell, frequency of cells showing micronuclei, and nuclear area were significantly increased in tobacco users than controls, especially in combined tobacco users. Nuclear-cytoplasmic ratio was increased and cytoplasmic area was decreased in tobacco users than controls.

**Conclusion:**

Tobacco in any consumable form is genotoxic. Smoking and smokeless tobacco, when consumed together, synergistically causes higher genetic damage. Different tobacco habits have different deleterious effects on oral mucosa, and these effects are more pronounced when the patients have combined habits. So, detecting the genotoxic changes through exfoliative cytology can be used as a simple yet reliable marker for early detection of carcinogenesis.

## Introduction

Oral cancer is one of the top three cancers in India accounting for 30% of all the cancers. The most widespread form of oral cancer mainly depends of tobacco consumption in any form, which is closely associated not only with the development of oral cancer, but also with a poor prognosis (Kashyap et al., 2012). The most aggressive chemicals present in tobacco cause extensive genetic damage to the human body, some of which are irreversible. Genetic damage gets started long before the clinical lesion appears. So, early diagnosis and prevention is very essential. Buccal cells, being the first barrier, represent a preferred target site for early genotoxic events induced by carcinogenic agents through inhalation or ingestion route and are capable of metabolizing proximate carcinogens to reactive products (Torres-Bugarin et al., 2014). These changes include formation of micronuclei, and alterations in nuclear size, cell size, nuclear cytoplasmic ratio, nuclear shape, nuclear discontinuity, optical density and nuclear texture. Exfoliative cytology could be of great value for identifying these genotoxic changes. The present study was undertaken to assess these genotoxic changes like micronuclei frequency, nuclear area, cytoplasmic area and the nuclear-cytoplasmic ratio of the squames from clinically normal buccal mucosa of tobacco users (smokers, tobacco chewers and combined habit group) and non-users of tobacco and to compare and correlate the findings.

## Materials and Methods

Institutional ethical clearance was obtained before commencing the study. A total number of 120 individuals without oral lesions were included in the study.

• Group I - Individuals habituated with smoking tobacco - 30

• Group II - Individuals habituated with smokeless tobacco - 30

• Group III - Individuals habituated with both smoking and smokeless tobacco - 30

• Group IV - Individuals without any deleterious habits – 30 (Controls)

Individuals with any history of systemic diseases and recent history of any viral infection or hospitalization, recent exposure to radiologic investigations, habituated with alcohol were excluded from the study.

Smears were taken by scraping the buccal mucosa of the participants gently in relation to premolar-molar area with the use of wooden spatula. The smears were taken in pre-cleaned, number coded microscopic slides and were fixed in 70% ethanol. Four smears were collected from each individual. All the smears were stained using Papanicolaou stain (PAP) using the manufacturer recommended protocol provided in the Rapid PAP kit. Feulgen staining was done using the protocol mentioned by (Gopal & Padma, 2018). All the PAP and Feulgen-stained slides were viewed under light microscope and cytomorphometric analysis was done with the help of Jenoptik pRogress software. Hundred cells per patient were evaluated for micronuclei using the criteria mentioned by (Tolbert et al., 1992). The extra nuclear cytoplasmic DNA fragments satisfying the following criteria were counted as micronuclei. 

• Micronuclei must be clearly separated from the main nucleus. 

• Micronuclei must have a smooth, oval or round shape. 

• Texture similar to nucleus. 

• Less than a third the diameter of associated nucleus, but large enough to discern shape and color. 

• Staining intensity similar to nucleus. 

• Same focal plane as nucleus. 

The criteria for excluding cells for micronuclei assessment by (Tolbert et al., 1992) were also followed. The cells with the following features were not taken for micronuclei assessment: 

• Cells with two nuclei.

• Dead or degenerating cells (karyolysis, karyorrhexis, nuclear fragmentation). 

• Nuclear blebbings (micronucleus- like structure connected with the main nucleus with a bridge). 

• Anucleated cells.

The mean number of micronuclei, mean micronuclei per cell, frequency of cells showing micronuclei were evaluated for each patient. Cytomorphometric assessment of nuclear area, cytoplasmic area and nuclear-cytoplasmic area was done for 100 cells in each patient using Jenoptik pRogress software tools. Results obtained were analysed using one-way ANOVA, Kruskal Wallis test, and Mann Whitney test followed by post-hoc tests.

## Results

Results obtained were similar using either PAP or Feulgen stain in almost all the parameters evaluated. The mean number of micronuclei, mean micronuclei per cell, and frequency of cells showing micronuclei were significantly higher in tobacco users (Groups I, II and III) when compared with controls (Group IV). Among the participants habituated to tobacco, all the parameters related to micronuclei were highest in the combined tobacco users (Group III) followed by smokeless tobacco users (Group II), and smokers (Group I) (p<0.001) (Table 1). 

Cytomorphometric assessment of nuclear area, cytoplasmic area (Cell area - Nuclear area), and nuclear-cytoplasmic ratio was done using Papanicolaou stain. Mean nuclear area was significantly higher in tobacco users when compared with controls. Among the habits groups, nuclear area was significantly increased in smokers followed by smokeless tobacco users and combined tobacco users (p<0.001) (Table 2). Comparison of mean cytoplasmic area and nuclear-cytoplasmic ratio using one-way-ANOVA showed no significant difference among the various study groups. 

**Table 1:** Intergroup Comparisons of Mean Number of Micronuclei, Mean Micronuclei Per Cell, Frequency of Cells Showing Micronuclei among Various Study Groups Using Feulgen Stain

Mean Number of Micronuclei		
Group (Mean ± SD)	Compared with	p-value
Group I (7.13±2.45)	Group II (10.30±4.16)	0.014*
Group I (7.13±2.45)	Group III (15.77±6.12)	<0.001*
Group I (7.13±2.45)	Group IV (0.60±1.19)	<0.001*
Group II (10.30±4.16)	Group III (15.77±6.12)	<0.001*
Group II (10.30±4.16)	Group IV (0.60±1.19)	<0.001*
Group III (15.77±6.12)	Group IV (0.60±1.19)	<0.001*
Mean Micronuclei per cell		
Group (Mean ± SD)	Compared with	p-value
Group I (1.172±0.312)	Group II (1.201±0.243)	1.000
Group I (1.172±0.312)	Group III (1.469±0.405)	0.022*
Group I (1.172±0.312)	Group IV (0.305±0.525)	<0.001*
Group II (1.201±0.243)	Group III (1.469±0.405)	0.051
Group II (1.201±0.243)	Group IV (0.305±0.525)	<0.001*
Group III (1.469±0.405)	Group IV (0.305±0.525)	<0.001*
Frequency of cells showing Micronuclei		
Group (Mean ± SD)	Compared with	p-value
Group I (14.27±4.891)	Group II (20.60±8.324)	0.014*
Group I (14.27±4.891)	Group III (31.53±12.247)	<0.001*
Group I (14.27±4.891)	Group IV (1.20±2.384)	<0.001*
Group II (20.60±8.324)	Group III (31.53±12.247)	<0.001*
Group II (20.60±8.324)	Group IV (1.20±2.384)	<0.001*
Group III (31.53±12.247)	Group IV (1.20±2.384)	<0.001*

†, Kruskal Wallis, post hoc. two sided P.value ≤ 0.05

**Figure 1:**
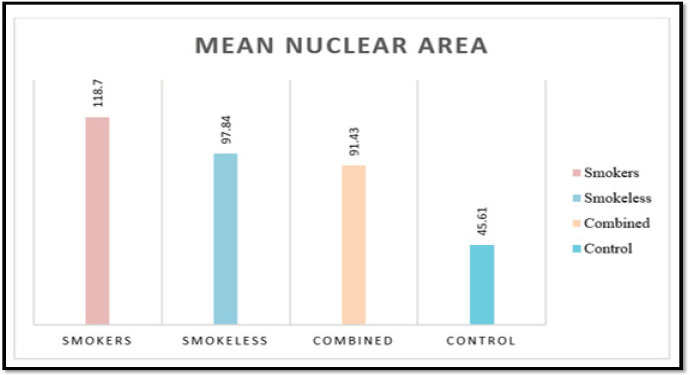
Comparison of Mean Nuclear Area among the Study Groups Using Pap Stain

**Table 2:** Intergroup Comparison of Mean Nuclear Area among the Different Groups Using Pap Stain

Group (Mean ± SD)	Compared with	p-value
Group I (118.7±22.38)	Group II (97.84±15.28)	<0.001*
Group I (118.7±22.38)	Group III (91.43±21.04)	<0.001*
Group I (118.7±22.38)	Group IV (45.61±18)	<0.001*
Group II (97.84±15.28)	Group III (91.43±21.04)	1
Group II (97.84±15.28)	Group IV (45.61±18)	<0.001*
Group III (91.43±21.04)	Group IV (45.61±18)	<0.001*

**Figure 2:**
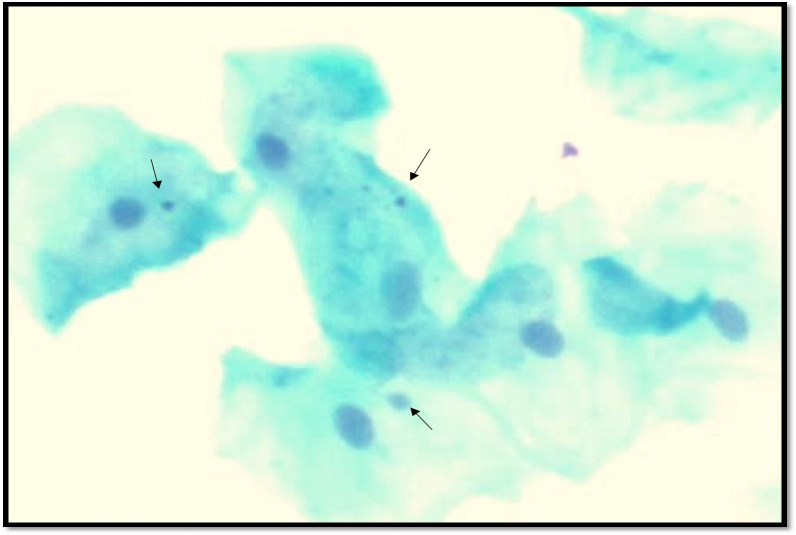
Smears Stained with Feulgen Stain Showing Micronuclei (Arrows)

**Figure 3:**
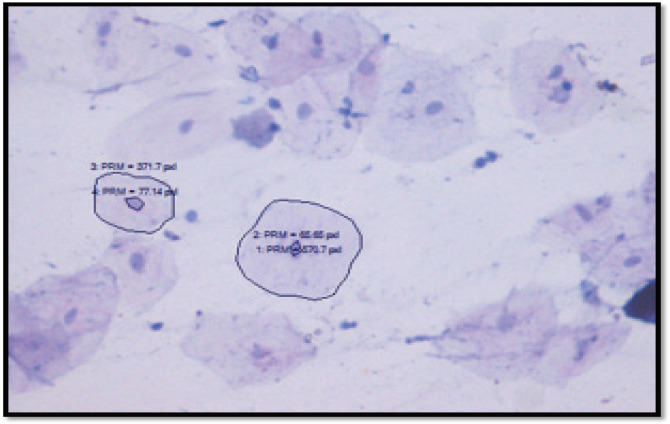
Cytomorphometric Analysis of Nuclear Area, Cytoplasmic Area using Pap Stain

## Discussion

Oral cancer is a multistage disease; it arises from normal mucosa, progresses to dysplasia and ultimately ends as cancer. Development of oral cancer proceeds through discrete genetic changes that occurs due to loss of genomic integrity after the continuous exposure to carcinogenic agent (Park et al., 2011).

The carcinogenic effect of the tobacco habits inducing genotoxic effect on oral mucosal cells can be found with investigations. It is widely accepted that genotoxic studies in exfoliated buccal cells remains one of the reliable sensitive markers in early diagnosis of oral cancer in tobacco users (Singam et al., 2019). Our study assesses the genotoxic effect of different types tobacco on the oral mucosa before the lesions appear in the oral cavity. Our study was designed to evaluate micronuclei and cytomorphometric changes (nuclear area, cytoplasmic area, nuclear-cytoplasmic ratio) associated with smokers, smokeless tobacco users, combined tobacco users and healthy individuals without any habits. Buccal smears from all subjects in the four study groups were stained with PAP and Feulgen stain and the parameters were evaluated.

We observed that the mean number of micronuclei and mean micronuclei per cell were increased in combined tobacco users than smokers and smokeless tobacco users. Our results were in accordance with the studies conducted by (Sellapa et al., 2009; Dash et al., 2018). On the other hand, our findings were contradictory to the findings observed by (Bonaasi et al., 2003; Pradeep et al., 2014) who stated that the number of micronuclei was increased in smokers than other groups. We also found that frequency of cells showing micronuclei was significantly increased in combined tobacco users when compared with other groups. Similar findings were observed by (Upadhyay et al., 2019; Chandirasekar et al., 2019). The micronuclei related genotoxic alterations in the cells may be due to the possibility that the buccal mucosa cells get direct exposure to the carcinogenic amines present in the tobacco (Proia et al., 2006). The cells bearing the damaged DNA will mostly survive and replicate with the damage and result in higher frequency of micronuclei (Moghaddam et al., 2020). Tobacco specific nitrosamines are believed to be responsible for the induction of micronuclei (Muhammed et al., 2021). Increase in all the micronuclei related changes may be due to the synergistic effect of combined use of smoking and smokeless tobacco which results in a higher genotoxicity in buccal mucosa cells than when they are consumed alone (Dash et al., 2018). Heat and chemical exposure from smoking and continuous exposure of tobacco specific amines while taking the smokeless tobacco prevents the cells from further dividing and in turn the nuclei get disintegrated due to the carcinogenic exposure and induces the formation of micronuclei. Smoking and smokeless tobacco when consumed together has been associated with increased risk of oral squamous cell carcinoma (Mello et al., 2019). Individuals with the habit of smoking and alcohol together are more prone to oral cancers than those to have the habits separately (Liu et al., 2015).

We found that the smokers group showed the highest mean nuclear area when compared to smokeless tobacco users and combined tobacco users. Similar findings were observed by (Einstein et al., 2005; Khot et al., 2015). Tobacco causes increase in nuclear size of the buccal cells. It is due to cellular adaptation of the cell in response to the carcinogens in the tobacco. Buccal epithelial cells have a decreased turn over and they will be in cell cycle for long periods which in turn increases the nuclear area. We found that mean cytoplasmic area is significantly higher in control group when compared to tobacco users group. Similar findings were observed by (Parmar et al., 2010; Babuta et al., 2014; Santos et al., 2017;). Decrease in the cytoplasmic area of smokeless tobacco users may be due to the fact that there is a close contact between the smokeless tobacco and oral mucosa. It causes the carcinogenic by-products to infiltrate into the mucosa since it will be kept in the oral cavity for longer period. As a result, the cell undergoes dehydration and causes the shrinkage of cytoplasm. We also found that the nuclear-cytoplasmic ratio was significantly higher in smokeless tobacco group when compared to smokers and controls. Similar findings were observed by (Singh et al., 2014; Khot et al., 2015). The increase in nuclear cytoplasmic area in smokeless tobacco might be due to the synchronous increase in the nuclear area and decrease in cytoplasmic area. Our results were in accordance with the study conducted by (Parmar et al., 2015; Mohan et al., 2017)

There are limited studies which evaluate the micronuclei and cytomorphometric changes in clinically normal appearing oral mucosa in comparison with different type of tobacco users. To the best of our knowledge, this is the first study to compare the cytotoxic effects of smoking tobacco users, smokeless tobacco users and combined tobacco users in clinically healthy mucosa. 

We observed that all parameters related to micronuclei were increased in the habits groups. It was the highest in the combined users group, which suggests that the synergistic effect of smoking and smokeless tobacco could cause greater genomic damage. Smokers group, however, showed pronounced alterations in cytomorphometric parameters, especially the nuclear area. Smokeless tobacco users had an elevated nuclear cytoplasmic ratio, suggesting that individuals with smokeless tobacco habit show both nuclear alterations, and changes in cytoplasmic area. Based on the findings of our study, we conclude that different tobacco related habits have different deleterious effects on the buccal mucosal cells, and these effects are more pronounced when the patients have both types of habits together.

## Author Contribution Statement

Study conception and design - Kokila Sivakumar and Harikrishnan Prasad. Clinical studies and sample collection - Kokila Sivakumar. Data acquisition and analysis - Kokila Sivakumar. Statistical analysis - Harikrishnan Prasad. General supervision - Srichinthu Keniyan Kumar, Rajmohan Muthusamy, Mahalakshmi Loganathan, Shanmuganathan Sivanandham, Prema Perumal. Manuscript preparation - Kokila Sivakumar and Harikrishnan Prasad. Manuscript editing and suggestions – Harikrishnan Prasad and Srichinthu Keniyan Kumar. All the authors have equal contribution in the study and manuscript works. All of them reviewed the results and approved the final version of the manuscript.
